# The use of expired resuscitation medications for life-threatening first aid conditions: a systematic search and narrative review^☆^

**DOI:** 10.1016/j.resplu.2025.101110

**Published:** 2025-09-23

**Authors:** Nathan Charlton, David C. Berry, Vijay Kannan, Ryan Yee, Jestin N. Carlson, Aaron M. Orkin

**Affiliations:** a University of Virginia, Department of Emergency Medicine, USA; b Department of Kinesiology, Saginaw Valley State University, USA; c MD Program, Temerty Faculty of Medicine, University of Toronto, Toronto, Canada; d Department of Emergency Medicine, Allegheny Health Network, USA; e Department of Family and Community Medicine, University of Toronto, Toronto, Canada

**Keywords:** Medication, Expired, First aid, Albuterol, Salbutamol, Aspirin, Epinephrine, Naloxone

## Abstract

**Introduction:**

First aid providers may encounter life-threatening conditions requiring treatment with medications. Given that resuscitation medications in first aid kits may be administered infrequently, first aid providers may face situations where only expired medications are available.

**Objective:**

This systematic search with a narrative review aims to evaluate the efficacy and safety of expired life-saving medications commonly used in first aid.

**Methods:**

We conducted a search of PubMed, EMBASE, Web of Science, CINAHL, and Cochrane Library (inception–April 2025) for studies regarding expired albuterol, epinephrine, aspirin, or naloxone. Two reviewers independently screened titles and abstracts, followed by full-text reviews to determine eligibility. We included randomized controlled trials (RCTs), clinical trials, systematic reviews, meta-analyses, and observational studies evaluating expired medications’ potency and safety. Data extraction focused on study design, population, interventions, comparators, outcomes, and key findings.

**Results:**

After deduplication, 1398 records were screened, and 17 studies met inclusion criteria: albuterol (*n* = 2), aspirin (*n* = 4), epinephrine (*n* = 8), and naloxone (*n* = 3). Albuterol (salbutamol) retained 98 % active drug 20–30 years past expiration. Aspirin (acetylsalicylic acid) could retain active drug for up to 40 years after expiration. Epinephrine autoinjectors could retain epinephrine for at least 36 months after expiration. Naloxone retained active drug for at least 19 months after expiration. There was minimal evidence of harmful degradation products.

**Conclusions:**

Under individual study conditions, the evaluated expired first aid medications maintained active drug and were largely free of harmful byproducts beyond their labeled expiration dates. Scientific and ethical principles may suggest possible benefits from expired medications in emergency settings when alternatives are unavailable.

## Introduction

First aid (FA) providers are called to care for life-threatening conditions in which treatment with resuscitation medications can be lifesaving. Common medications and emergencies that anyone may encounter include albuterol for respiratory distress, aspirin for non-traumatic chest pain, epinephrine for anaphylaxis, and naloxone for opioid poisoning.^1^, ^2^ While administration of an unexpired medication is preferred, there are situations and settings when FA providers are faced with life-threatening scenarios and only expired medications are available. In these situations, knowledge of the potential risks and benefits of administering expired resuscitation medications is critical.

Expiration dates are established based on stability testing by manufacturers to ensure that medications retain their full potency and safety up to that date.^3^ The dates are indicators of the time during which the manufacturer guarantees the full potency and safety of a drug. Expiration dates do not necessarily mean that the drug becomes ineffective or harmful after this date. Some medications are known to remain stable and retain most of their potency for years beyond their expiration dates.^4^, ^5^, ^6^, ^7^ Large-scale regulatory studies and shelf-life extension programs (SLEPs) play an essential role in evaluating physical and chemical properties of drugs to promote the safe extension of drug expiration dates^8^ though these studies do not immediately address the questions about real-world use when only expired products might be accessible. The potential risks of using expired medications include reduced efficacy due to decreased potency and the administration of unwanted degradation products.^6^, ^7^, ^9^ However, in life-threatening emergencies, the benefit of administering an expired resuscitation medication, with the hope of some retained efficacy, may outweigh these risks, especially when no other options are readily available.

As part of its continual evaluation process, the American Red Cross Scientific Advisory Council (ARC SAC) performed a review on this topic to inform guidelines for the use of expired medications in first aid settings. This systematic search and narrative review aims to evaluate the evidence on the efficacy and safety of expired resuscitation medications commonly used in life-threatening first aid conditions.

## Methods

A protocol for this review was developed prior to the conduct of the review, internal to the ARC SAC, and included a review question, preliminary search strategy developed with the assistance of a medical librarian, inclusion and exclusion criteria, a plan for risk of bias assessment, and a plan for data extraction and analysis.

### Search strategy

A comprehensive literature search was performed initially on March 4, 2020, as part of an initial ARC SAC review, subsequently on January 22, 2024, as part of a triennial review, and additionally re-run on April 12, 2025, using PubMed, EMBASE, Web of Science, CINAHL, and Cochrane Library Clinical Trials (Cochrane CENTRAL). The search terms included “albuterol,” “aspirin,” “epinephrine,” “naloxone,” and associated terms for expiration dates and drug stability. The search covered publications from database inception to April 2025. The detailed search strategy is provided in Appendix A.

### Eligibility criteria

While FA encompasses a wide range of conditions, we focused on those for which early pharmacologic intervention may be most beneficial, including self-administration and administration by lay FA providers. We chose the list of FA medications by consensus among the authors to include albuterol (salbutamol) for asthma exacerbations, aspirin for non-traumatic chest pain, epinephrine for anaphylaxis, and naloxone for opioid overdose. We limited our search to these medications because they address common, time-sensitive sentinel conditions where critical decisions about expired medication use could have immediate impacts on morbidity and mortality. These medications constitute a unique, non-exhaustive set of pharmaceuticals with demonstrated resuscitative application in the first aid context, and that are commonly included in general first aid education and first aid kits.^10^

We included randomized controlled trials (RCTs), clinical trials, systematic reviews, meta-analyses, and observational studies evaluating expired medications' potency and safety. For this study, retained potency was assessed as the amount of active ingredient detected in a product after its labeled expiry date with the assumption that active ingredients retain the same biologic activity delineated from original FDA approval or long-term stability studies. Our results included published manuscripts as well as scientific meeting abstracts. Studies were limited to those published in English, as this was the language spoken and understood by reviewers. We excluded animal studies, non-English publications, and studies without outcome measures directly relevant to medication pharmacology (e.g. medication volume over time).

### Data extraction and quality assessment

Two reviewers (NC and JNC) independently screened titles and abstracts, followed by full-text reviews to determine eligibility. Data extraction focused on study design, population, interventions, comparators, outcomes, and key findings. Given the anticipated heterogeneity of the data, we planned to perform a narrative review and did not plan to perform a meta-analysis. All authors contributed to discussion on the final papers to include until consensus through email or meeting. Two reviewers (NC and RY) completed the risk of bias (RoB) assessment using the Office of Health Assessment and Translation (OHAT) RoB tool,^11^ which assessed bias across 6 domains using 11 structured questions and 4 ordinal responses: definitely low, probably low, probably high, or definitely high risk of bias. This tool was selected for its capacity to evaluate various bodies of evidence, including exposure and observational data from human, animal, and in vitro studies.^11^, ^12^

## Results

**Fig. 1:**
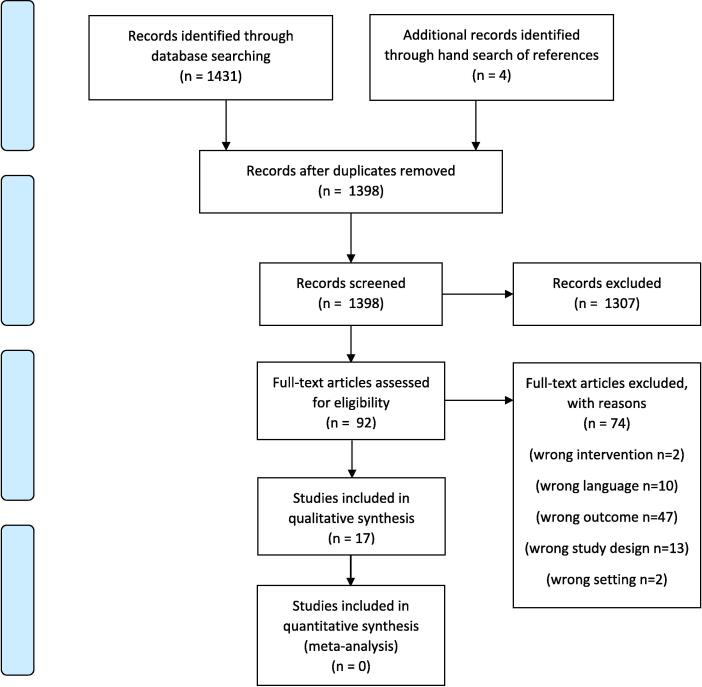
PRISMA flow diagram.

Of the 1435 records identified, 1398 were screened after deduplication and 17 studies were included in the final review, examining albuterol (*n* = 2),^7^, ^13^ aspirin (*n* = 4),^4^, ^14^, ^15^, ^16^ epinephrine (*n* = 8),^5^, ^17^, ^18^, ^19^, ^20^, ^21^, ^22^, ^23^ and naloxone (*n* = 3),^6^, ^24^, ^25^ (Fig. 1). These are summarized in Table 1. Studies excluded at the full text level can be found in Appendix B.

**Table 1:** Characteristics of Included Studies.

**Author**	**Year**	**Medication**	**Study Design**	**Population**	**Key Findings**
Albuterol (Salbutamol)					
Zilker M, et al.^7^	2019	Salbutamol sulfate, Salbutamol	Bench research	50 medications, 5 samples of Salbutamol (albuterol) sulfate, 1 sample of Salbutamol, 20–30 + years old were studied to evaluate their content and degradation profiles.	The content of salbutamol was above 98 % in all samples. Impurities were present, but in all cases were less than 0.5 %
Kutty, R et al.^13^	2022	Albuterol (nebulized), Albuterol (MDI) montelukast	Bench research (abstract only)	“Expired lots of albuterol that were donated by a local free clinic” No dates reported.	Of the samples tested, 80 % if the nebulized albuterol and 40 % of the MDI albuterol retained > 90 % of their potency.
Aspirin (Acetylsalicylic acid/ASA)					
Thomis R, et al.^14^	1984	Aspirin combination tablets containing Aspirin, acetaminophen, and ascorbic Acid	Bench research	Tablets containing 300 mg aspirin, 200 mg acetaminophen, and 300 mg ascorbic acid were aged 4–61 months to study degradation effects. Two tablets were used for each test.	Initial reported concentration of Aspirin in each tablet ranged from 80.9-99.2 % and generally degraded over time. The two lowest Aspirin concentrations were at 45 months (76.3 %) and 55 months (80.9 %). At 61 months the salicylic acid (breakdown product) concentration was 9.4 %
Verstraeten A, et al.^15^	1987	Aspirin tablets	Bench research	Aspirin tablets aged between 6–59 months were studied for degradation effects, no living subject population was used.	Aspirin content found in the 14 different brands of ASA tabs from analysis by HPLC ranged from 83.9 % to 102.2 % and varied by tablet age. Only two tablet brands were outside of the claimed validity period (36 months) but were each found to have > 100 % [101.8 % (at 40 months) and 101.3 % (at 50 months), respectively] of their claimed ASA content. Both tablets also had a low salicylic acid breakdown product (0.59 % and 0.18 %, respectively).
Cantrell L, et al.^4^	2012	Aspirin combination tablets	Bench research	Three tablets of two different aspirin combination tablets, which were between 28–40 years expired. Specific dates not listed.	Aspirin was found present in less than 12 % of its labeled concentration in both combinations tested: Combo pill #1 (Fiorinal): declared aspirin content 200 mg; mean measured aspirin content 2.28 mg (SD 0.10); Combo pill #2 (Codempiral): declared aspirin content 226.8 mg; mean measured content 1.53 mg (SD 0.04)
Wotring VE^15^	2016	Aspirin	Bench research	Medications were part of a supply kept at the international space station for 550 days. Five tested aspirin tablets were 9 months post expiration date.	Tablets were found to contain active ingredients within 96.5 % of label claim of 325 mg per tablet (mean 313.7, SD 0.05). Free salicylic acid was less than 0.05 % in each sample.
Epinephrine					
Simons KJ, et al.^17^	2000	Epinephrine autoinjectors	Bench research	Twenty-eight epinephrine autoinjectors (EAIs) and 6 pediatric EAIs that were between 1–90 months post expiration date.	There was a greater amount of epinephrine content (mg) between in-date adult EAIs (0.326 ± 0.003) and outdated adult EAIs (0.238 ± 0.008) and (MD 0.088; 95 % CI 0.08–0.09) and between in-date pediatric EAIs (0.148 ± 0.007) and outdated pediatric EAIs (0.108 ± 0.011) and (MD 0.04; 95 % CI 0.03–0.05). There was a negative correlation of 0.63 between epinephrine content and the number of months since expiration.
Simons KJ, et al.^18^	2011	Epinephrine autoinjectors	Bench research (abstract only)	Six 0.15 EAIs 129 months past expiration date, six 0.15 mg EAIs 151 months past expiration date, six 0.3 mg autoinjectors 130 months past expiration date.	The mean epinephrine dose ejected from the 0.15 EAIs was 60.1 % (mg not reported) at 129 months post expiration and 54.7 % (mg not reported) at 151 months. The mean dose ejected from the 0.3 mg EAI was 77.1 % at 130 months post expiration.
Simons KJ, et al.^19^	2012	Epinephrine autoinjectors	Bench research (abstract only)	Thirty-five EAIs (0.15 mg and 0.3 mg) that were 3 to 36 months past expiration date. Epinephrine content was measured using HPLC-UV.	Epinephrine doses decreased with increasing months past expiry date (no correlation coefficient reported). EAIs that were ≤ 24 months past expiry date ejected ≥ 90 % of E doses (mg not reported).
Rachid O, et al.^20^	2015	Epinephrine autoinjectors	Bench Research	Thirty 0.3 mg EAIs and five 0.15 mg EAIs, between 3 to 36 months past expiration date and kept under uncontrolled storage conditions were collected in an allergy practice.	Epinephrine concentrations ranged from 84.2 % to 101.5 % of reported values. There was a correlation between the number of months past the expiration date and decreasing concentration of epinephrine in the solution. (R^2^ = 0.779). All EAIs ≤24 months past labeled expiration dating contained ≥90 % of the USP labeled epinephrine concentration.
Cantrell FL, et al.^21^	2017	Epinephrine autoinjectors	Bench research	Thirty-one 0.3 mg EAIs and nine 0.15 mg EAIs, 1–50 months past their expiration dates	Nineteen 0.3 mg (61 %) and five 0.15 mg (56 %) autoinjectors contained ≥ 90 % of reported epinephrine content. All of the expired EAIs tested contained at least 80 % of their labeled epinephrine concentration and decrease in content percentage correlated with time.
Weir WB ^22^	2018	Epinephrine prefilled syringes for injection	Bench research	Six epinephrine prefilled syringes, 1 mg/10 mL, were stored in a climate-controlled setting for 30 months after expiration.	There was no statistical difference between the epinephrine concentrations in the expired products tested compared with the unexpired control. There were no degradation products detected, and no bacterial or fungal growth occurred.
Kassel L, et al.^5^	2019	Epinephrine autoinjectors	Bench research	Forty-six EAIs (0.15 & 0.30 mg; breakdown not reported) that were 1–168 months past their expiration dates	Approximately 80 % (*n* = 37) of the EAIs retained ≥90 % of stated epinephrine potency, 9 contained <90 %. The median amount of drug remaining in expired EAIs was 97.2 %, IQR of 8.1 %. EAIs up to 6 months past expiration date (*n* = 4) retained 100 % concentration, EAIs 1 year (*n* = 10) retained at least 95 % concentration, EAIs (*n* = 27) up to 30 months retained at least 90 % drug content.
Saleheen A, et al.^23^	2020	Epinephrine autoinjectors	Bench research	23 EAIs (unknown dose) that were expired from 12-207 months were tested by ultra-high-pressure liquid chromatography to determine concentration.	Epinephrine concentrations in the expired EAIs in the range of 0.05–1.43 mg/mL. The typical concentration of epinephrine in autoinjectors is 1 mg/mL. There was a correlation of *r* = −0.37 between epinephrine concentration and months since expiration.
Naloxone					
Lyon RC, et al.^24^	2006	Naloxone HCl	Bench research	Naloxone HCl inj soln tested as part of the US Food and Drug Administration shelf-life extension program	All 10 lots of naloxone HCl were extended at least 5 years past their expiration date based on data from the shelf-life extension program.
Pruyn S, et al.^6^	2019	Naloxone HCl	Bench research	11 samples of expired (2–27.5 years) naloxone injection. Samples were either pre-filled syringes or ampules. These were compared against non-expired products.	10 samples retained more than 90 % of naloxone up to 27 years after expiration. 90. Only one sample (25.5 years exp) had a concentration less than 90 % (89 %). The concentration of nornaloxone was less than 1 % in all samples and primarily in detected in older samples.
Hossain MF, et al.^25^	2022	Naloxone HCl	Bench research	Four lots of naloxone injections (*n* = 3) and naloxone nasal spray (*n* = 3) that were between 6–19 months post expiration.	The naloxone concentration was 102.8 % +/- 2.55 of expected for the nasal spray and mean of 105.9 % +/- 1.25 for the injection. No degradation products were identified.

**Study Characteristics** Table 1 summarizes the characteristics of the included studies.

### Albuterol (Salbutamol)

Two bench research studies were identified pertaining to albuterol suggesting that albuterol retains efficacy past expiration under controlled conditions^7^, ^13^ (Table 1). Kutty et al.^13^ found that most donated albuterol samples retained over 90 % of their active drug well beyond their expiration dates. Zilker et al.^7^ evaluated the stability of albuterol 20–30 years past expiration and found that it retained 98 % of its stated active drug, with impurities being less than 0.5 % of the overall content.

### Acetylsalicylic acid (Aspirin)

Four bench research studies were identified pertaining to aspirin^4^, ^14^, ^15^, ^16^ (Table 1). Wotring^16^ found that aspirin tablets that were 9 months post expiration date retained 96.5 % of their reported active drug. Cantrell et al.^4^ found that combination tablets containing aspirin had less than 90 % of their labeled active drug after 28–40 years, but this reduction was within expected degradation ranges. Cantrell et al.’s^4^ study also noted that while aspirin content was reduced, the degradation was not significantly harmful and still within safe limits for use, without clinically relevant degradation products. Two additional studies, Thomis et al.^14^ and Verstraeten et al.,^15^ found that while the amount of acetylsalicylic acid in non-expired tablets decreased over time, the medication retained substantial active drug without identified degradation products.

### Epinephrine

Eight studies were identified pertaining to epinephrine^5^, ^17^, ^18^, ^19^, ^20^, ^21^, ^22^, ^23^ (Table 1). All eight contained information from bench research. Simons et al.^17^ demonstrated that there was a greater amount of epinephrine content (mg) between in-date adult EAIs (0.326 ± 0.003) and outdated adult EAIs (0.238 ± 0.008) (MD 0.088; 95 % CI 0.08–0.09) and between in-date pediatric EAIs (0.148 ± 0.007) and outdated pediatric EAIs (0.108 ± 0.011) and (MD 0.04; 95 % CI 0.03–0.05). Simons et al. ^17^ demonstrated 54.7 % of the reported active drug at 151 months in 0.15 mg EAI and 77.1 % in 0.3 mg EAIs at 130 months post expiration. Simons et al. ^18^ and Rachid et al. ^19^ and both reported that all EAIs tested ≤24 months post expiration retained >90 % of the expected dose. Cantrell et al.^20^ further supported these findings, demonstrating that all epinephrine concentrations in autoinjectors remained above 80 % for up to 50 months. Finally, Kassel et al.^5^ reported that all autoinjectors tested retained >90 % drug content up to 30 months their expiration dates.

### Naloxone

Three studies were identified that used laboratory analysis to determine the concentration of naloxone in expired products^6^, ^24^, ^25^ (Table 1). Hossain et al.^25^ found that naloxone that was expired by up to 19 months retained >100 % mean active drug. Pruyn et al.^6^ confirmed that naloxone solutions maintained high amounts of active drug after up to 27 years, with minimal formation of nornaloxone, a potential degradation product. The FDA's Shelf-Life Extension Program (SLEP) study by Lyon et al.^24^ extended the expiration dates of naloxone by 5 years, supporting its long-term stability.

### Quality assessment and risk of bias

Of the 17 studies included, risk of bias was generally low across the 6 domains assessed with the OHAT Tool (Appendix C). In the domain of selection bias, studies employing convenience samples,^5^ varying storage conditions,^19^, ^20^ and no control groups for comparison^16^ were evaluated as probably high risk of bias. Observational studies that co-assessed potential contaminants and degradation products had lower risk of confounding bias.^5^, ^22^ Few studies reported blinding procedures,^23^ though performance and detection biases were often definitely low or probably low risk due to strong exposure characterization methods and similarity between experimental conditions across study groups. Attrition/exclusion and selective reporting biases were low across full-text studies with most papers including complete outcome data. Only the observational data from epinephrine studies such as Simons et al.,^17^ Simons et al.,^18^ Simons et al.,^19^ and Saleheen et al.,^23^ was directly related to our study question.

## Discussion

The findings of this review suggest that select resuscitation medications used in the first aid setting retain concentrations of active drug for months to years after the listed expiration date. Furthermore, of the resuscitation medications tested, there was no evidence of harmful breakdown products. In emergencies, FA providers often face unpredictable circumstances that require immediate action; the primary concern of FA providers is to stabilize the patient and prevent deterioration of their condition until professional medical help is available. In such cases, when no other options are available, the potential benefits of using expired medications may outweigh the risks associated with reduced efficacy.

While medications can degrade over time and with suboptimal storage conditions, our work has shown that certain medications can retain active ingredients at their labeled concentration past their expiration date with minimal impurities and contaminants. Manufacturers are required by the United States Food and Drug Administration (FDA) and other equivalent regulatory bodies to establish an expiration date for their products and submit stability data to support that expiration date.^3^ Expiration dates typically range up to three years, however, many medications may retain >90 % of their base potency well beyond this expiration period.^24^ Harm from toxic degradation byproducts has rarely been associated with use of expired medications (e.g., few reports about specific tetracycline byproducts^26^) and shelf-life extension programs affirm many emergency preparedness medications can be used after expiry.^24^, ^27^ We believe the primary concern of using expired resuscitation medications is reduced efficacy during lifesaving interventions due to lack of active drug or lack of administration. While fears of regulatory violation and myths about efficacy of expired medications may persist and affect use, prior regulatory studies have shown that many drugs retain a significant percentage of their potency even years after expiration.^28^, ^29^, ^30^ For instance, the study conducted by the U.S. Food and Drug Administration (FDA) on stockpiled medications found that about 90 % of more than 100 prescription and over-the-counter drugs were safe and effective far beyond their original expiration date.^24^ In light of shortages, Health Canada has recommended that if you are experiencing an anaphylactic reaction and only have an expired EpiPen, you should use it immediately and seek emergency medical care.^31^ Although some drug products may be more prone to degradation under certain conditions, making them less suitable candidates for shelf-life extension, expired resuscitation medications could still have potential to provide lifesaving benefits, and may be beneficial in instances were no unexpired alternatives are available.

### Albuterol (salbutamol)

Albuterol is used in emergencies to relieve bronchospasm in asthma exacerbations or other respiratory distress conditions. Albuterol, also termed salbutamol, is a Beta-2 agonist available as a metered-dose inhaler (MDI) and dry powder inhaler (DPI).^32^ While expired albuterol may not be as potent, its use could still help open the airways, potentially providing enough relief to prevent clinical deterioration until the patient can receive further medical attention. Stability of albuterol has been explored previously. In a study by Zilker et al.,^7^ albuterol was found to retain almost 100 % of its original active drug after more than 20 years past its expiration dating. Degradation products were found after 20 years; however, these products were found in low concentrations and were nontoxic.^7^

### Aspirin

Acetylsalicylic acid (aspirin) is a first-line treatment for myocardial infarction because of its antiplatelet effects, which can reduce vascular and ischemic events and death.^33^ While likely not as time sensitive as other medications used in first aid, early administration of aspirin compared with later administration has been demonstrated to improve outcomes.^34^ When expired aspirin is the only available option, administering it may still provide some benefit in delaying disease progression until more definitive care can be provided. Given the recommendation to chew 160–325 mg of ASA during an acute myocardial infarction,^33^ and considering that expired aspirin products may contain less ASA than non-expired formulations, administration of a non-expired 162 mg dose may be considered when it becomes available if only 162 mg of expired aspirin was initially used.

### Epinephrine

Epinephrine is critical in emergency settings for individuals experiencing anaphylaxis. Rapid-onset allergic reaction can lead to airway obstruction, cardiovascular collapse, and death if not treated promptly. Epinephrine is an Alpha-1, Beta-1, and Beta-2 adrenergic receptor agonist that is the drug of choice for the emergency treatment of severe allergic reactions and anaphylaxis.^35^ Cantrell et al.^4^ found that expired epinephrine auto-injectors still retained active medication even years after expiration, though at a reduced level. These studies demonstrated that, over time, epinephrine loses potency and becomes discolored, but there has been no evidence of harmful degradation products found in expired epinephrine solution. Even after being heated or frozen to extreme temperatures, as long as EAIs are able to function, they were found to retain a portion of their original epinephrine content.^17^, ^21^, ^36^, ^37^^.^ This indicates that expired EAIs, even stored under suboptimal conditions, may still retain lifesaving medication. In a life-threatening situation where no other alternatives exist, administering an expired epinephrine injection could potentially save a life by providing at least some degree of vasoconstriction and bronchodilation, which are critical actions in the treatment of anaphylaxis.

### Naloxone

Naloxone is an opioid receptor competitive antagonist used emergently to reverse a life-threatening opioid overdose.^38^ In this review, naloxone was shown to retain its potency years after its expiration dating. In a study completed by the FDA shelf-life extension program, 10 different expired lots of naloxone HCl were evaluated for potency, stability, impurities, preservatives, appearance, and pH. Expiration dates of all lots of evaluated naloxone were extended by the FDA for an additional 5 years.^24^ One potentially harmful degradation product of naloxone, nornaloxone (noroxymorphone), was detected in samples of naloxone that had been stored >20 years past their expiration dates, though this product was not found in clinically significant amounts. While nornaloxone may antagonize the effects of naloxone, it would not be expected to produce toxicity outside of the opioid toxidrome. As naloxone appears to be stable after years of storage, the use of an expired product in an emergent setting appears beneficial.

The decision to use expired resuscitation medications is not without ethical and practical considerations. Medical professionals and FA providers are generally advised against using expired drugs due to concerns about reduced efficacy and potential liability issues. However, when the patient's life is at risk and no other options are available, the ethical principle of beneficence (doing good) may justify using expired medications.^39^ Furthermore, the likelihood of expired medications causing harm in many emergencies is significantly lower than the harm posed by doing nothing. While FA providers and organizations may consider maintaining stocks of critical emergency medications and routinely checking and replacing them before expiration whenever possible, this is not always feasible in remote or resource-limited settings, making the judicious use of expired medicines necessary. In low-resource settings, the decision to use expired medications can be particularly complex due to limited access to healthcare resources, including the availability of fresh medicines. One of the primary advantages of using expired medications in low-resource settings is the potential to save lives when no viable alternatives are available or readily accessible. In such cases, using expired medications can provide critical care that might prevent severe outcomes. This knowledge can empower first aid providers and organizations to significantly impact challenging situations.

The use of expired FA medications has drawbacks. The most pressing concern is the potential for reduced efficacy. While many medications retain some potency after expiration, the degree to which they remain effective can vary, and relying on them may lead to suboptimal patient outcomes. Furthermore, time is not the only consideration in these situations. The same factors that lead to infrequent stocking in low-resource settings may also lead to improper storage conditions such as extremes of temperature, which are also known to impact efficacy and breakdown. Standard guidelines on using expired medications are lacking. This ambiguity can confuse healthcare providers regarding when and how to use these drugs safely, potentially compromising patient safety. When used for non-emergent purposes in low-resource settings, expired medications can pose ethical and legal challenges. Medical providers may face liability issues if patients suffer harm due to ineffective treatment with expired drugs. Additionally, the reliance on expired medications might perpetuate systemic inequities in healthcare, as those in low-resource settings are more likely to face such risks, which could further widen the health disparities gap.^39^

### Limitations

This review has limitations, including the variability in study designs and storage conditions, which may affect the generalizability of the results, but also captures the complex and myriad scenarios that providers in low-resource settings often face. Future research should focus on standardized methods for evaluating the stability of expired medications under various environmental conditions. Furthermore, no studies evaluated the bioavailability of the expired medications in humans, which is not too surprising given the previously discussed ethical and legal implications of administering expired medications. While it was assumed that if medications retained some active ingredient amounts after expiration then there was potential for efficacy, the evidence gathered could not explore whether the studied drugs may exhibit complex, non-linear pharmacodynamic/dose–response relationships that could result in paradoxical or idiosyncratic effects in humans. Future studies may also yield variations on results that relate to shelf-life extensions occurring after this study. We did not specifically assess whether included studies had stereoselective power to differentiate between enantiomeric forms of active drug ingredients. This has been discussed in Cantrell^40^ with respect to L- and D- epinephrine, where it was concluded that despite racemization, the benefits of using an expired EpiPen for anaphylaxis likely outweigh the risks associated with pursuing no treatment. Albuterol has been classically provided as a racemic solution (equal amounts of R/S-albuterol) for over 50 years,^41^ and racemization of naloxone and ASA is less relevant due to their chemical structures. Expanding the range of medications studied will provide a more comprehensive understanding of the potential for using expired drugs in emergency settings. We explored albuterol, naloxone, ASA, and epinephrine because of their lifesaving properties and relevance across diverse care contexts, including lay and standard first aid, first response, and emergency medical practice settings. How these results generalize to other medicines is unknown. Another medication with similar lifesaving potential we did not assess was expired glucose for otherwise indicated hypoglycemia, acknowledging that non-expired sources of effective glucose could be accessible from non-pharmaceutical sources outside first aid kits. While some studies used drugs sampled from tightly controlled settings (e.g., climate-controlled facilities,^22^ others used samples from uncertain or unique environments (e.g., donated medications^13^ and even the International Space Station^16^), and this variability could be a relative strength that promotes external validity, as retained active ingredients and the absence of harmful degradation products was consistently observed across studies.

## Conclusion

In this review examining four resuscitation medications used in the first aid setting, albuterol, aspirin, epinephrine, and naloxone maintained active ingredient at or near their product label after their expiration date. Furthermore, no studies found significant amounts of harmful byproducts. While these results do not assure potency or efficacy, they suggest that in certain first aid emergencies, life-saving medications may offer a favorable benefit to risk ratio even when they are past their expiration date. Further research might help determine the medication-specific efficacy duration after the expiration date. This review may help to inform decisions regarding the use of these expired resuscitation medications in the first aid setting when balancing the potential benefits and risks that the expired medication holds in life-threatening circumstances when no readily available alternatives exist.

## CRediT authorship contribution statement

**Nathan Charlton:** Writing – review & editing, Writing – original draft, Methodology, Investigation, Formal analysis, Conceptualization. **David C. Berry:** Writing – review & editing, Writing – original draft, Methodology, Investigation, Formal analysis, Conceptualization. **Vijay Kannan:** Writing – review & editing, Writing – original draft, Methodology, Investigation, Formal analysis, Conceptualization. **Ryan Yee:** Writing – review & editing, Writing – original draft, Formal analysis. **Jestin N. Carlson:** Writing – review & editing, Writing – original draft, Methodology, Investigation, Formal analysis, Conceptualization. **Aaron M. Orkin:** Writing – review & editing, Writing – original draft, Supervision, Methodology, Investigation, Formal analysis, Conceptualization.

## Funding

This research did not receive any specific grant funding. NC, DCB, VK, JNC and AMO receive travel compensation from the American Red Cross Scientific Advisory Council for attendance at meetings.

## Declaration of competing interest

The authors declare that they have no competing financial or personal interests. NC, DCB, VK, JNC and AMO are members of the American Red Cross Scientific Advisory Council.

## Figures and Tables

**Table:** 

**Database**	**Search strategy**
Pubmed	(“Epinephrine”[Mesh] OR epinephrine OR adrenaline OR “Aspirin”[Mesh] OR aspirin OR “acetylsalicylic acid” OR 2-acetyloxybenzoic acid OR “Naloxone”[Mesh] OR naloxone OR narcan OR evzio OR “Glucagon”[Mesh] OR glucagon OR “Albuterol”[Mesh] OR albuterol) AND (“expiration date” OR “expiry date” OR “sell by date” OR “end of use” OR “use by date” OR “shelf life” OR “beyond use” OR (“Drug Stability”[Mesh] AND (“time factors”[Mesh] OR expiration OR expiry OR expire OR expired OR “expiration date” OR “expiry date” OR “sell by date” OR “end of use” OR “use by date” OR “shelf life” OR “beyond use”)))
Embase	('epinephrine'/exp OR 'epinephrine autoinjector'/exp OR 'epinephrine' OR 'acetylsalicylic acid'/exp OR 'aspirin' OR 'naloxone'/exp OR 'naloxone' OR 'glucagon'/exp OR 'glucagon' OR 'salbutamol'/exp OR 'albuterol') AND ('expiration date'/exp OR expiration OR expiry OR expire OR expired OR 'expiration date' OR 'expiry date' OR 'sell by date' OR 'end of use' OR 'use by date' OR 'shelf life' OR 'beyond use')
CINAHL	((MH Epinephrine + ) OR epinephrine OR adrenaline OR (MH Aspirin+) OR aspirin OR “acetylsalicylic acid” OR “2-acetyloxybenzoic acid” OR (MH Naloxone + ) OR naloxone OR narcan OR evzio OR (MH Glucagon+) OR glucagon OR (MH Albuterol + ) OR albuterol) AND (“expiration date” OR “expiry date” OR “sell by date” OR “end of use” OR “use by date” OR “shelf life” OR “beyond use” OR ((MH “Drug Stability+”) AND ((MH “time factors+”) OR expiration OR expiry OR expire OR expired OR “expiration date” OR “expiry date” OR “sell by date” OR “end of use” OR “use by date” OR “shelf life” OR “beyond use”)))
Cochrane Library, Clinical Trials (Cochrane CENTRAL	([mh Epinephrine] OR epinephrine OR adrenaline OR [mh Aspirin] OR aspirin OR “acetylsalicylic acid” OR “2-acetyloxybenzoic acid” OR [mh Naloxone] OR naloxone OR narcan OR evzio OR [mh Glucagon] OR glucagon OR [mh Albuterol] OR albuterol) AND (“expiration date” OR “expiry date” OR “sell by date” OR “end of use” OR “use by date” OR “shelf life” OR “beyond use”)
Web of Science	(TI=(((epinephrine OR adrenaline OR aspirin OR “acetylsalicylic acid” OR “2-acetyloxybenzoic acid” OR naloxone OR narcan OR evzio OR glucagon OR albuterol) AND (“expiration date” OR “expiry date” OR “sell by date” OR “end of use” OR “use by date” OR “shelf life” OR “beyond use”)))) OR AB=(((epinephrine OR adrenaline OR aspirin OR “acetylsalicylic acid” OR “2-acetyloxybenzoic acid” OR naloxone OR narcan OR evzio OR glucagon OR albuterol) AND (“expiration date” OR “expiry date” OR “sell by date” OR “end of use” OR “use by date” OR “shelf life” OR “beyond use”)))

**Table:** 

## Appendix B Excluded studies

1. Drugs past their expiration date. *Med Lett Drugs Ther.* 2009;51(1326):100–101.
2. Drugs past their expiration date. *Med Lett Drugs Ther.* 2015;57(1483):164–165.
3. Drugs past their expiration date. Med Lett Drugs Ther 2020;62(1603):117–119.
4. Drugs for the doctor's bag: 1—adults. Drug Ther Bull. 2005;43(9):65–68. https://doi.org/10.1136/dtb.2005.43965.
5. Storage and shelf-life of drugs: when is it important? Drug Ther Bull. 1977;15(21):81–3.
6. Abe T, Yanagihara Y, Uchino T, Oriyama T, Komatsu M, Nakajima K, Suzuki H. Evaluation of the pharmaceutical characteristics of various enteric-coated aspirin tablets under different storage conditions. Chem Pharm Bull (Tokyo). 2014;62(7):617–26. https://doi.org/10.1248/cpb.c13-00580.
7. Agosti S, Qu P, Brown J. The effects of heating on EpiPen epinephrine auto-injector device integrity and function. *J Allergy Clin Immunol.* 2020;145(2):AB10. https://doi.org/10.1016/j.jaci.2019.12.859
8. Anderson BD, Darrington RT. Influence of temperature and hydrophobic group-associated icebergs on the activation energy of drug decomposition and its implication in drug shelf-life prediction. Pharm Res. 1991;8(5):661–2. https://doi.org/10.1023/a:1015877328588.
9. Asker AF, Whitworth CW. Evaluation of dimethylpolysiloxane fluids as vehicles for various pharmaceuticals. I: effect on stability and dissolution of aspirin. J Pharm Sci. 1974;63(10):1630–1. https://doi.org/10.1002/jps.2600631033.
10. Atíes DAM, Peña CMG, Porto YMDO, et al. Stability of a formulation of 1 mg/mL adrenaline injectable product for veterinary use. Rev Cubana Farm. 2015;49(3).
11. Bodie B, Brodell RT, Helms SE. Shortage of lidocaine with epinephrine: Causes and solutions. J Am Acad Dermatol. 2018;79(2):392–393. https://doi.org/10.1016/j.jaad.2018.02.035. Epub 2018 Mar 1.
12. Bonhomme L, Benhamou D, Comoy E, Preaux N. Stability of epinephrine in alkalinized solutions. Ann Emerg Med. 1990 Nov;19(11):1242–4. https://doi.org/10.1016/s0196-0644(05)82281–4.
13. Boswell B, Brown J. A systematic review of the concentration of epinephrine in auto-injectors after device expiration. J Allergy Clin Immunol. 2020;145(2):AB3.
14. Brown, J.C., Cooper, A.Q., Parish, H.G., & Qu, P. (2021). The Effects of Heating on EpiPen Epinephrine Auto-injector Device Integrity and Function. J. Allergy Ther;12(2): No:233.
15. Carstensen JT. Stability of solids and solid dosage forms. J Pharm Sci. 1974;63(1):1–14. https://doi.org/10.1002/jps.2600630103.
16. Carstensen JT, Attarchi F, Hou XP. Decomposition of aspirin in the solid state in the presence of limited amounts of moisture. J Pharm Sci. 1985 Jul;74(7):741–5. https://doi.org/10.1002/jps.2600740709. PMID: 4032246.
17. Ceschel GC, Badiello R, Ronchi C, Maffei P. Degradation of components in drug formulations: a comparison between HPLC and DSC methods. J Pharm Biomed Anal. 2003 Aug 8;32(4–5):1067–72. https://doi.org/10.1016/s0731-7085(03)00210–3. PMID: 12899995.
18. Chang P, Arulappu R. Biological activity of “expired” adrenaline. J Pharm Pharmacol. 1974;26(10):712–713.
19. Chatain C, Boccon-Gibod I, Bouillet L, Leccia MT. Protracted severe food anaphylaxis: Should we incriminate expired epinephrine pen or clonal mast cell disorder? Ann Allergy Asthma Immunol. 2018 Jan;120(1):109. https://doi.org/10.1016/j.anai.2017.08.292. PMID: 29273123.
20. Church WH, Hu SS, Henry AJ. Thermal degradation of injectable epinephrine. Am J Emerg Med. 1994 May;12(3):306–9. https://doi.org/10.1016/0735–6757(94)90145–7. PMID: 8179736.
21. Cooper A, Brown J, Qu P. The effects of freezing on epinephrine auto-injector device function. Ann Allergy Asthma Immunol. 2018;121(5):S57-S58.
22. Cuervas-Mons Vendrell M, Fernández Prieto M, Sánchez Sánchez MT, Maestre Fullana MA, Abad Lecha E, Salvador Palacios A, de Frutos Soto A. Posible validez de medicamentos termolábiles fuera de las condiciones de conservación recomendadas por el fabricante [Potential validity of thermolabile drugs outside the preservation conditions recommended by the manufacturer]. Farm Hosp. 2004 Nov-Dec;28(6):440–4. Spanish. PMID: 15628947.
23. Curtis C, Stukus D, Scherzer R. Epinephrine preparedness in pediatric patients with food allergy: an ideal time for change. Ann Allergy Asthma Immunol. 2014 Jun;112(6):560–2. https://doi.org/10.1016/j.anai.2014.04.009. PMID: 24860922.
24. De Winter S, Vanbrabant P, Vi NT, Deng X, Spriet I, Van Schepdael A, Gillet JB. Impact of temperature exposure on stability of drugs in a real-world out-of-hospital setting. Ann Emerg Med. 2013 Oct;62(4):380–387.e1. https://doi.org/10.1016/j.annemergmed.2013.04.018. Epub 2013 May 24. PMID: 23711878.
25. Du B, Daniels VR, Vaksman Z, Boyd JL, Crady C, Putcha L. Evaluation of physical and chemical changes in pharmaceuticals flown on space missions. AAPS J. 2011 Jun;13(2):299–308. https://doi.org/10.1208/s12248-011–9270-0. Epub 2011 Apr 9. PMID: 21479701; PMCID: PMC3085701.
26. Ebel F, Mastilovic O. Measurement of color change and expiration time determination in tablets by reflexion spectrophotometry. Acta Pharm Jugosl. 1974;24(1):37–41.
27. Fatima QUA, Hassan F, Farid Hasan SM, Israr F. Stability evaluation of different brands of aspirin tablets available in local market of Karachi, Pakistan. Lat Am J Pharm. 2015;34(7):1358–1363.
28. Fleury Souverain S, Sigrist T, Gschwind L, Bouchoud L, Sadeghipour F, Bonnabry P. Development of ready-to-use adrenaline syringes for emergency use. Eur J Hosp Pharm. 2014;21:A133.
29. Frey J, Pruyn S, Baker B, et al. Meeting the demand for naloxone: stability of expired naloxone solution. Clin Toxicol (Phila). 2018;56(10):917.
30. Fyllingen G, Langvik TA, Hasselgård P, Roksvaag PO. Racemization and oxidation in adrenaline injections. Acta Pharm Nord. 1990;2(5):355–62.
31. Gairard AC, Demirdjian S, Vasseur R, Renard G, Chast F. Stabilité de l'adrénaline dans des solutions pour irrigations intra-oculaires [Epinephrine stability in solutions for intra-ocular irrigations]. J Fr Ophtalmol. 1996;19(12):743–7. French.
32. Gebhart F. Do expiration dates make a difference? Drug Top. 2005;149(19).
33. Gracia-Vásquez SL, Gracia-Vásquez YA, Villanueva-Ramírez TJ, et al. Public practice regarding disposal of expired and unused medication. Lat Am J Pharm. 2019;38(8):1687–1695.
34. Gucluyildiz H, Banker GS, Peck GE. Determination of porosity and pore-size distribution of aspirin tablets relevant to drug stability. J Pharm Sci. 1977 Mar;66(3):407–14. https://doi.org/10.1002/jps.2600660323.
35. Hakes LB, Corby TC, Meakin BJ. The stability of salbutamol solution [proceedings]. J Pharm Pharmacol. 1979;31:25P.
36. Heeb RM, Stollhof B, Reichhold J, Thiesen J, Krämer I. Stability of ready-to-administer and ready-to-use epinephrine and norepinephrine injection solutions. Pharm Technol Hosp Pharm. 2018;2(4):159–171.
37. Hoellein L, Holzgrabe U. Ficts and facts of epinephrine and norepinephrine stability in injectable solutions. Int J Pharm. 2012 Sep 15;434(1–2):468–80. https://doi.org/10.1016/j.ijpharm.2012.05.017.
38. Ibrić S, Jovanović M, Djurić Z, Parojcić J, Solomun L, Lucić B. Generalized regression neural networks in prediction of drug stability. J Pharm Pharmacol. 2007;59(5):745–50. https://doi.org/10.1211/jpp.59.5.0017.
39. Iserson KV. Should We Use Expired Drugs When Necessary? J Emerg Med. 2021;60(5):669–673. https://doi.org/10.1016/j.jemermed.2021.02.002.
40. Kerddonfak S, Manuyakorn W, Kamchaisatian W, Sasisakulporn C, Teawsomboonkit W, Benjaponpitak S. The stability and sterility of epinephrine prefilled syringe. Asian Pac J Allergy Immunol. 2010;28(1):53–7.
41. Kersten D, Göber B. Zur Auswertung nichtisothermer Kurzzeittests von Arzneistofflösungen [The evaluation of non-isothermal short-term tests of drug solutions]. Pharmazie. 1984;39(8):541–6. German.
42. Khedr T, Ryad L, Youssef AO. Testing the validity of reference standard materials and stock solutions of veterinary drugs using LC-MS/MS. Food Addit Contam Part A Chem Anal Control Expo Risk Assess. 2019;36(3):405–412. https://doi.org/10.1080/19440049.2019.1568585.
43. Lombardelli S, Edgar D. Adrenaline auto-injectors are not renewed within their expiration period. *Clin Exp Allergy.* 2009;39(12):1947.
44. Lopez AB, Gaig Jané P, Gázquez García V, Dalmau Duch G. Use and handling of self-injectable epinephrine in patients with a history of anaphylaxis. Allergy. 2011;66:642.
45. Marriage D. An audit of the prescription and use of adrenaline auto-injector devices in adults and children. Clin Exp Allergy. 2017;47(12):1685.
46. McCarthy P. Shelf life explained [2]. Pharm J. 1999;262(7026):18.
47. McCray A, Brown J, Qu P. The effects of washing on EpiPen epinephrine auto-injector device integrity and function. J Allergy Clin Immunol. 2020;145(2):AB5.
48. Mennickent S, Yates T, Vega M, Gloria Godoy C, Saelzer R. Estudio de la estabilidad química de comprimidos de ácido acetilsalicílico mediante un control de estanterías en farmacias de Concepción, Chile [Study of the chemical stability of acetylsalicylic acid tablets during storage in pharmacies of Concepción, Chile]. Rev Med Chil. 2002;130(4):409–15. Spanish..
49. Mihranyan A, Strømme M, Ek R. Influence of cellulose powder structure on moisture-induced degradation of acetylsalicylic acid. Eur J Pharm Sci. 2006;27(2–3):220–5. https://doi.org/10.1016/j.ejps.2005.10.002.
50. Mollica JA, Ahuja S, Cohen J. Stability of pharmaceuticals. J Pharm Sci. 1978;67(4):443–65. https://doi.org/10.1002/jps.2600670405.
51. Murthy KS, Ghebre-Sellassie I. Current perspectives on the dissolution stability of solid oral dosage forms. J Pharm Sci. 1993;82(2):113–26. https://doi.org/10.1002/jps.2600820202.
52. Norwood DL, Paskiet D, Ruberto M, Feinberg T, Schroeder A, Poochikian G, Wang Q, Deng TJ, DeGrazio F, Munos MK, Nagao LM. Best practices for extractables and leachables in orally inhaled and nasal drug products: an overview of the PQRI recommendations. Pharm Res. 2008;25(4):727–39. https://doi.org/10.1007/s11095-007–9521-z.
53. Pierson JC. Let's put an expiration date on the current approach to drug expiration dates. J Am Acad Dermatol. 2014;71(1):193–4. https://doi.org/10.1016/j.jaad.2013.10.068.
54. Prescott JD. Considerations for the pharmacist about anaphylaxis and epinephrine auto-injectors. Pharmacy Times. 2014;80(8).
55. Puente Y, Daza JC, Monteseirin FJ. Evaluation of the ability to use a self-administered epinephrine injector. Allergy. 2015;70:487.
56. Rachid O, Simons FE, Rawas-Qalaji M, Lewis S, Simons KJ. Epinephrine doses delivered from auto-injectors stored at excessively high temperatures. Drug Dev Ind Pharm. 2016 Jan;42(1):131–135. https://doi.org/10.3109/03639045.2015.1035283.
57. Rak J, Chalabala M. Stabilita kyseliny acetylsalicylove v liekových formách [Stability of acetylsalicylic acid in dosage forms]. Cesk Farm. 1971;20(4):154–60. Slovak.
58. Rawas-Qalaji M, Simons FE, Collins D, Simons KJ. Long-term stability of epinephrine dispensed in unsealed syringes for the first-aid treatment of anaphylaxis. Ann Allergy Asthma Immunol. 2009;102(6):500–3. https://doi.org/10.1016/S1081-1206(10)60124-X.
59. Rawas-Qalaji MM, Rachid O, Simons FE, Simons KJ. Long-term stability of epinephrine sublingual tablets for the potential first-aid treatment of anaphylaxis. Ann Allergy Asthma Immunol. 2013;111(6):568–70. https://doi.org/10.1016/j.anai.2013.09.005.
60. Rusznak C. The importance of patients regularly and visually checking their epinephrine auto-injectors. Asim Allerji Immunoloji. 2018;16(3):179–181.
61. Sakkal E, Bitar Y, Trefi S. Quality control of the active pharmaceutical ingredients of some pharmaceutical products prior to the termination of their shelf life. Res J Pharm Technol. 2019;12(12):6111–6118.
62. Schaber P. Monitoring of expiry data for use of drugs without computer: the 5-color system. Dtsch Apoth Ztg. 1985;125(31):1572–1573.
63. Špringer V, Sopková A. On the problems related to unused drugs 1. Questionnaire survey of pharmacy customers. Farm Obzor. 2001;70(11):283–287.
64. Stewart AG. Expiry dates on drug packages [2]. Trop Doct. 1997;27(3):191.
65. Storms WW, Tringale M, Ferro TJ. The impact of expired and empty quick-relief asthma inhalers: The Asthma and Allergy Foundation of America's Asthma Inhaler Design Survey. Allergy Asthma Proc. 2015;36(4):300–5. https://doi.org/10.2500/aap.2015.36.3854.
66. Storms WW, Tringale M, Waldron A. Allergy and Asthma Foundation of America's Asthma Inhaler Design (ID)™ survey: use of outdated and empty quick-relief inhalers remains a problem. Ann Allergy Asthma Immunol. 2013;111(5):A40.
67. Turner R. Improving drug stability & drug delivery by modification of MDI canister surfaces. ONdrugDelivery. 2015;(62):33–35.
68. Wark H, Sara C. The stability of adrenaline injection 1 in 10000. Br J Anaesth. 1983 Nov;55(11):1139–40. https://doi.org/10.1093/bja/55.11.1139.
69. Welter, C.; Roschel, K.; Schneider, S.; Marson, C.; Stammet, P. Refer impact of ambient temperature on 5 emergency drugs aboard an emergency medical car over a 1-year period. Annals of Emergency Medicine 2022;80(4):358–363.
70. Whitfield D, Bosson N, Habrat D, Telmos J, Watson G, Eckstein M, Kazan C, Gausche-Hill M. Critical medication shortages: effective strategies to maintain continuous medication availability for emergency medical services. Prehosp Emerg Care. 2020;24(2):290–296. https://doi.org/10.1080/10903127.2019.1619001.
71. Wright JP, Codini M, Craig A, et al. Loss of epinephrine auto-injector time-to-expiration at purchase: a hidden premium on cost. Acad Emerg Med. 2018;25:S132.
72. Wyttenbach N, Birringer C, Alsenz J, Kuentz M. Drug-excipient compatibility testing using a high-throughput approach and statistical design. Pharm Dev Technol. 2005;10(4):499–505. https://doi.org/10.1080/10837450500299875.
73. Zilker M, Sörgel F, Holzgrabe U. A systematic review of the stability of finished pharmaceutical products and drug substances beyond their labeled expiry dates. J Pharm Biomed Anal. 2019;166:222–235. https://doi.org/10.1016/j.jpba.2019.01.016.
74. Zilker M, Sörgel F, Holzgrabe U. Surprisingly stable: analysis of old medicines indicates considerably longer shelf lives. Dtsch Apoth Ztg. 2019;159(18).
